# It is difficult to tell if there is a Condorcet spanning tree

**DOI:** 10.1007/s00186-016-0535-3

**Published:** 2016-02-06

**Authors:** Andreas Darmann

**Affiliations:** Institute of Public Economics, University of Graz, Graz, Austria

**Keywords:** Spanning tree, Condorcet, Computational complexity, Social choice theory

## Abstract

We apply the well-known Condorcet criterion from voting theory outside of its classical framework and link it with spanning trees of an undirected graph. In situations in which a network, represented by a spanning tree of an undirected graph, needs to be installed, decision-makers typically do not agree on the network to be implemented. Instead, each of these decision-makers has her own ideal conception of the network. In order to derive a group decision, i.e., a single spanning tree for the entire group of decision-makers, the goal would be a spanning tree that beats each other spanning tree in a simple majority comparison. When comparing two dedicated spanning trees, a decision-maker will be considered to be more satisfied with the one that is “closer” to her proposal. In this context, the most basic and natural measure of distance is the usual set difference: we simply count the number of edges the spanning tree has in common with the proposal of the decision-maker. In this work, we show that it is computationally intractable to decide (1) if such a spanning tree exists, and (2) if a given spanning tree satisfies the Condorcet criterion.

## Introduction

Dating back more than two centuries (Condorcet 1785), the Condorcet criterion is one of the fundamental and most important concepts in voting theory (see Brams and Fishburn 2002 for a survey). Originally formulated for single winner elections, the criterion states that, if an alternative exists that wins^1^ by a simple majority against each other alternative in a pairwise comparison, that alternative should be declared the winner of the election. The Condorcet criterion has been generalized to the election of committees in several ways, e.g., by Fishburn (1981), Gehrlein (1985), Kaymak and Sanver (2003), and more recently by Elkind et al. (2014) and Darmann (2013a).

In the last decades, researchers have started to use the Condorcet criterion outside of its classical framework, often under the label of *popularity*. In particular, this has been done in the context of a popular matching in a bipartite graph, i.e., a matching which beats each other matching in the graph in a simple majority comparison (see, e.g., Gärdenfors 1975). In that respect, a lot of works are concerned with the computational complexity involved in finding such a popular matching, examples being the works of Abraham et al. (2005), Biró et al. (2010), and Huang and Kavitha (2011). In another recent work, Zuylen et al. (2014) consider the problem of finding a popular ranking from a computational viewpoint. In their framework, given a set of alternatives, each member of a set of agents proposes her ideal solution, i.e., individual ranking of the alternatives. The goal is to find a ranking $\pi$ meeting the property that there is no other ranking $\pi '$ which beats $\pi$ in a simple majority comparison. In Zuylen et al. (2014), these comparisons are performed by measuring the distance of the considered ranking to each of the agents’ rankings by means of the Kendall-tau distance.

We apply the Condorcet criterion to spanning trees of an undirected graph. Finding spanning trees in an undirected graph is a central task in operations research, with numerous applications in the construction of physical networks such as telecommunication networks, power transmission lines, and pipeline or highway systems (see also Ahuja et al. 1993). In this paper, we consider the situation in which such a network needs to be installed. Typically, however, the corresponding decision-makers involved do not agree on the network. Instead, each of them decides on her own ideal solution, i.e., spanning tree, which she proposes. Clearly, each of the decision-makers would like to see her ideal spanning tree being implemented, which is impossible except in the trivial case of all decision-makers proposing the same spanning tree. Now, it is natural to assume that, when considering two dedicated spanning trees, each of the decision-makers will be more happy with the one that is “closer” to her proposal. The most basic and natural measure of distance in this context is to simply count the number of edges the spanning tree has in common with the proposal of the decision-maker, i.e., to consider the usual set difference. Finally, to decide on an overall solution for the whole group of decision-makers, the well-studied Condorcet criterion is a meaningful and plausible criterion.

As an illustrative example we consider the member states of the European Union deciding on the layout of a communication network (e.g., a high security connection of law enforcement agencies) or a crucial supply network such as a gas or oil pipeline. These networks are generally designed as spanning trees connecting all countries represented by vertices in a graph. The decision-makers could be the countries themselves, or board members of some EU institution. For political and historical reasons involving trust, self-interest, or stability concerns, different decision-makers have different perceptions which links (i.e., edges) between the countries should be used in order to establish the whole network. In particular, the decision-makers could have different conceptions of the entire network because of political relations and alliances (especially in case the decision-makers are the countries themselves), or due to concerns regarding the security of supply and political stability. In addition, different decision-makers could base their conceptions on different criteria, which typically results in differing networks. Finding a plausible solution which takes into account the interests of all the decision-makers in such a setting is clearly an important aggregation task.

In principle, we are thus concerned with the aggregation of spanning trees of an undirected graph into a single spanning tree. Hence, this work is somewhat related to the axiomatically motivated work of Endriss and Grandi (2012), where, in a preference-based environment, each member of a set of agents proposes a directed graph, which need to be aggregated into a single graph. More formally, similar to the work of Zuylen et al. (2014), in our framework each member of a set of agents proposes her favourite solution, i.e., spanning tree of the graph. Now, a (weak) Condorcet tree is a spanning tree *T* such that for each other spanning tree $T'$, *T* beats (is not beaten by) $T'$ in a pairwise majority comparison. In order to perform these comparisons, we measure the distance between two spanning trees by means of the set difference. In this work, we consider two natural decision problems in connection with a (weak) Condorcet tree from a computational perspective. In the first one, we consider the question if such a spanning tree exists. In the second problem, we ask if a given specific spanning tree is a (weak) Condorcet tree.

The first question is related to the work of Darmann (2014), where instead of applying the Condorcet criterion, the goal is to find a spanning tree which does not deviate from each of the individual spanning trees by more than a given number of edges. Note that that problem is structurally rather different from the ones considered in this paper. For instance, a (weak) Condorcet tree might be identical with a particular spanning tree proposed by many agents while it might be completely disjoint from some other proposed spanning trees. Darmann (2014), however, proves that it is $\mathsf NP$-complete to decide if such a tree exists. In this work, by providing $\mathsf coNP$-hardness results we show that deciding if a (weak) Condorcet tree exists is also a computationally hard problem.

The second question is in the spirit of the seminal paper by Bartholdi et al. (1989), who show that both for the Dodgson and Kemeny voting rule it is $\mathsf NP$-hard to decide if a given candidate is a winner of an election. The related work of Darmann (2013b) focuses on the second question, in a different framework and using the terminology of popularity. In particular, Darmann (2013b) provides computational complexity results for the problem of deciding if a given spanning tree is popular^2^ with respect to scoring functions adopted from voting rules, with the focus on approval voting and Borda voting. The approval voting scenario of Darmann (2013b) is closely related to our approach, the main difference being that instead of whole spanning trees in Darmann (2013b) the agents propose single edges (which do not induce a certain structure) that should be included in the spanning tree. From Darmann (2013b) it follows that deciding if a given spanning tree is a weak Condorcet tree is $\mathsf coNP$-complete already if each of the agents propose two edges only. The computational complexity of the problem when the proposed edges form a certain structure, in particular a spanning tree of the given graph, is not investigated in Darmann (2013b) and, to the best of our knowledge, has not been studied before. In this work, we add to the result of Darmann (2013b) by showing that deciding if a given spanning tree is a (weak) Condorcet tree is $\mathsf coNP$-complete if the agents propose a whole solution, i.e., spanning tree, instead of single edges.^3^

## Problem definition

We briefly introduce the formal framework of this paper. An undirected graph $G=(V,E)$ consists of a finite set *V* and a set *E* of two-element subsets of *V*. We call the elements of *V* vertices and the elements of *E* edges. A subset $T\subseteq E$ with $|E|=|V|-1$ is called a *spanning tree* of *G*, if (*V*, *T*) contains no cycle. Note that for any two spanning trees $T_{1},T_{2}$ of *G* we have $|T_{1}\setminus T_{2}|=|T_{2}\setminus T_{1}|$.

Let $G=(V,E)$ be an undirected graph and let *A* be a set of agents. For each agent $a \in A$, we are given a spanning tree $T_{a}$ of *G*. Then, a spanning tree *T* of *G* is called a *Condorcet tree* if for all spanning trees $\tilde{T}$ of *G*, $\tilde{T} \not = T$,

$$\begin{aligned} \#\{a\in A:|T\setminus T_{a}|<|\tilde{T}\setminus T_{a}|\}>\#\{a\in A:|\tilde{T}\setminus T_{a}|<|T\setminus T_{a}|\} \end{aligned}$$

holds. Similarly, a spanning tree *T* of *G* is called a *weak Condorcet tree*, if for all spanning trees $\tilde{T}$ of *G*, $\tilde{T} \not = T$,

$$\begin{aligned} \#\{a\in A:|T\setminus T_{a}|<|\tilde{T}\setminus T_{a}|\} \ge \#\{a\in A:|\tilde{T}\setminus T_{a}|<|T\setminus T_{a}|\} \end{aligned}$$

is satisfied. Clearly, a Condorcet tree is also a weak Condorcet tree, but the reverse does not necessarily hold.

In the first decision problem considered in this paper we ask for the existence of a Condorcet tree.

### **Definition 1**

(Condorcet-Tree)

**Table:** 

GIVEN:	Set *A* of agents, undirected graph $G=(V,E)$, spanning trees $T_{a}$ of *G* for $a\in A$.
QUESTION:	Is there a Condorcet tree?

Analogously, Weak-Condorcet-Tree is the problem of deciding if there exists a weak Condorcet tree.

The second type of decision problem we consider is to decide if a given spanning tree is a (weak) Condorcet tree.

### **Definition 2**

(Condorcet-Tree Winner)

**Table:** 

GIVEN:	Set *A* of agents, undirected graph $G=(V,E)$, spanning trees $T_{a}$ of *G* for $a\in A$, distinguished spanning tree *T* of *G*.
QUESTION:	Is *T* a Condorcet tree?

Analogously, in Weak-Condorcet-Tree Winner we ask if a given spanning tree is a weak Condorcet tree.

Let $\omega :E\rightarrow \mathbb {N}_{0}$ be defined by $\omega (e):=|\{a\in A: e\in T_{a}\}|$. As captured by the lemma below, the candidates for a weak Condorcet tree – and hence for a Condorcet tree – can be restricted to the set of maximum spanning trees in *G* with respect to $\omega$, i.e., spanning trees in *G* that have the maximum weight with respect to $\omega$.

### **Lemma 1**

If *T* is a weak Condorcet tree, then *T* is a maximum spanning tree with respect to $\omega$.

### *Proof*

Assume that in the considered graph $G=(V,E)$, *T* is not a maximum spanning tree w.r.t. $\omega$. Then, by the path optimality condition for maximum spanning trees (cf. Ahuja et al. 1993) there must be an edge $e'=\{u,v\}\in E\setminus T$ such that $\omega (e')>\omega (e'')$ for some edge $e''$ contained in the unique path in *T* which connects the nodes *u* and *v*. Clearly, $T':=T\cup \{e'\}\setminus \{e''\}$ is again a spanning tree of *G*. Let $A_{T'}=\{a\in A:|T'\setminus T_{a}|<|T\setminus T_{a}|\}$ and $A_{T}=\{a\in A:|T\setminus T_{a}|<|T'\setminus T_{a}|\}$. Comparing $T'$ to *T* yields

$$\begin{aligned} \begin{array}{ccccc} |A_{T'}|-|A_{T}|= & {} \omega (e')-\omega (e'')> & {} 0\end{array}. \end{aligned}$$

Hence, *T* is not a weak Condorcet tree. $\square$

Lemma 1 is interesting in its own right as similar results have been stated for multiple referenda (Laffond and Lainé 2009) and committee selection (Brams et al. 2007a, b). In particular, Laffond and Lainé (2009) generalize a result of Brams et al. (2007a, b) which states that, using approval ballots and alternative-wise majority voting, the winning committee maximizes the total sum of the voters’ utilities when individual utility is measured by the number of alternatives included in the committee.

In what follows, we show that each of the problems Condorcet-Tree, Weak-Condorcet-Tree, Condorcet-Tree Winner, and Weak-Condorcet-Tree Winner is computationally hard.

## Computational intractability results

We start by showing that Condorcet-Tree is computationally intractable.

### **Theorem 1**

Condorcet-Tree is $\mathsf {coNP}$-hard.

### *Proof*

We provide a reduction from the $\mathsf coNP$-complete problem 3-Unsat, the complement of the classical $\mathsf NP$-complete problem 3-Sat. In an instance of 3-Unsat, we are given a set *X* of variables and a set *C* of (disjunctive) clauses made up of exactly 3 literals (a literal is a variable or the negation of a variable in *X*); the task is to decide if *C* is unsatisfiable, i.e., if there is no truth assignment for *X* that satisfies all the clauses in *C*. 3-Unsat remains $\mathsf coNP$-complete if the number of occurrences of each variable is bounded by 4 (3-Sat is known to be $\mathsf NP$-complete under these restrictions (Tovey 1984); obviously, 3-Unsat is $\mathsf coNP$-complete under the same restrictions).

Let $\mathcal {U}=(X,C)$ be such an instance of 3-Unsat, with $X=\{x_{1},\ldots ,x_{n}\}$ and $C=\{C_{1},\ldots ,C_{m}\}$ for some $n,m\in \mathbb {N}$. W.l.o.g. we assume that a clause does not contain both $x_{t}$ and $\bar{x}_{t}$ for some $t\in \{1,\ldots ,n\}$, since such a clause is satisfied by any feasible truth assignment. Let *Z* be the set of literals, i.e., $Z=\{x_{t},\bar{x}_{t}|\,1\le t\le n\}$. For $1\le j\le m$, we write $C_{j}=(z_{j_{1}}\vee z_{j_{2}}\vee z_{j_{3}})$ with $z_{j_{p}}\in Z$, $p\in \{1,2,3\}$. We identify a truth assignment $\phi$ with the set of literals set true under $\phi$.

*Step I: Constructing instance*$\mathcal {V}$*of *Condorcet*-*Tree*.* From $\mathcal {U}$, we construct an instance $\mathcal {V}$ of Condorcet-Tree as follows.

**Fig. 1:**
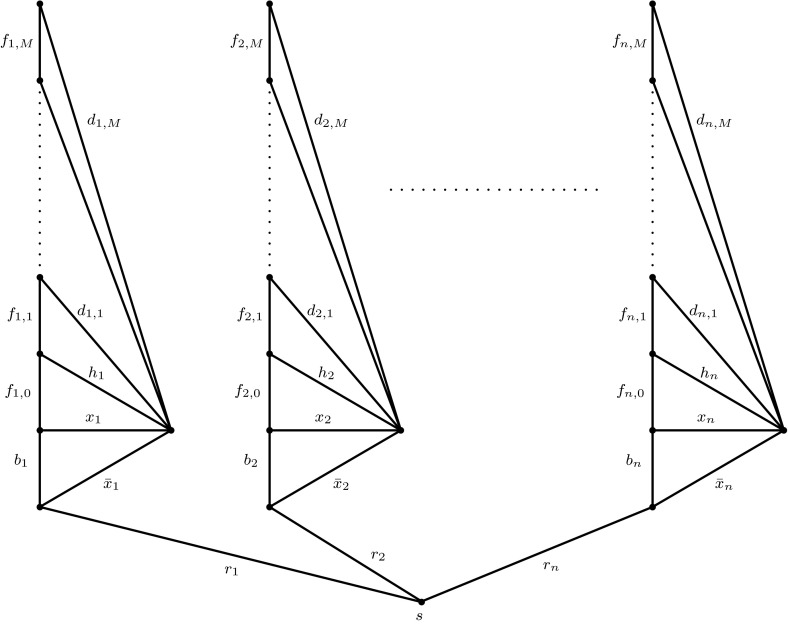
Graph *G* in instance $\mathcal {V}$ of Condorcet-Tree

*Step Ia: Defining the graph.* Let $M:=m+2n^{2}+5n+1$. First, in order to define a graph $G=(V,E)$ (see Fig. 1), we set

$$\begin{aligned} V:=\{s\}\cup \{s_{t},u_{t},v_{t},w_{t}|1\le t\le n\}\cup \{y_{t,q}|\,1\le t\le n,\,1\le q\le M\}. \end{aligned}$$

In addition, for $$1\le t\le n$$, we define the edges $$r_{t}=\{s,s_{t}\}$$, $$b_{t}=\{s_{t},u_{t}\}$$, $$x_{t}=\{u_{t},w_{t}\}$$, $$\bar{x}_{t}=\{s_{t},w_{t}\}$$, $$h_{t}=\{v_{t},w_{t}\}$$, and $$f_{t,0}=\{u_{t},v_{t}\}$$. Finally, for $$1\le t\le n$$, $$1\le q\le M$$, we define the edges $$d_{t,q}=\{y_{t,q},w_{t}\}$$ and $$f_{t,q}=\{y_{t,q-1},y_{t,q}\}$$, where $$y_{t,0}:=v_{t}$$. Let $$R:=\bigcup \{r_{t}\}$$, $$B:=\bigcup \{b_{t}\}$$, $$H:=\bigcup \{h_{t}\}$$$$D:=\bigcup \{d_{t,q}\}$$, and $$F:=\bigcup \{f_{t,q}\}$$, where the indices in the set unions run over all possible values of *t* and *q* respectively. Identifying a literal with an edge of the same label, we formally define

$$\begin{aligned} E:=R\cup B\cup D\cup F\cup H\cup Z. \end{aligned}$$

Note that $|V|=1+4n+nM$ and $|E|=6n+2nM$. Hence, any spanning tree $\tilde{T}$ of *G* consists of

1 $$\begin{aligned} |\tilde{T}|=|V|-1=n(M+4) \end{aligned}$$

edges.

*Step Ib: Introducing the agents. *We introduce a set $A=A_{H}\cup A_{D}\cup A_{C}\cup A_{Z}$ of agents.

The agent set $A_{H}$ consists of $m+2n^{2}+n$ agents, each $h\in A_{H}$ proposing the same spanning tree

$$\begin{aligned} T_{h}=T=R\cup B\cup F\cup H. \end{aligned}$$

For $1\le q\le M$, let $D_{q}:=\{d_{t,q}|1\le t\le n\}$. The next set of agents $A_{C}=\{\gamma _{j}|1\le j\le m\}$ is such that agent $\gamma _{j}$, in order to represent clause $C_{j}$, proposes the tree

$$\begin{aligned} T_{\gamma _{j}}:=R\cup B\cup F\cup \{z_{j_{1}},z_{j_{2}},z_{j_{3}}\}\cup (D_{j}\setminus \{d_{j_{1},j},d_{j_{2},j},d_{j_{3},j}\}). \end{aligned}$$

Next, we introduce the $2n^{2}+n$ agents in $A_{Z}$, where, for $1\le t\le n$ and $1\le q\le 2n+1$, agent $\zeta _{t,q}$ proposes the spanning tree

$$\begin{aligned} T_{\zeta _{t,q}}:=R\cup F\cup \{x_{t},\bar{x}_{t}\}\cup (B\setminus \{b_{t}\})\cup (D_{m+(t-1)\cdot (2n+1)+q}\setminus \{d_{t,m+(t-1)\cdot (2n+1)+q}\}). \end{aligned}$$

Finally, the agents in $A_{D}=\{\delta _{i}|1\le i\le 4n+1\}$ are such that $\delta _{i}$ proposes the spanning tree

$$\begin{aligned} T_{\delta _{i}}=R\cup B\cup F\cup D_{m+2n^{2}+n+i}. \end{aligned}$$

It is not hard to verify that each proposed solution is indeed a spanning tree of *G*. Note that

2 $$\begin{aligned} |A|=(m+2n^{2}+n)+m+(2n^{2}+n)+(4n+1)=2m+4n^{2}+6n+1 \end{aligned}$$

holds. In addition, observe that

- each edge in *H* is contained in the spanning tree *T* proposed by $m+2n^{2}+n$ agents,
- the set $R\cup F$ is contained in each of the proposed spanning trees,
- each edge in *B* is contained in all but $2n+1$ of the proposed spanning trees, while
- each edge in *D* is contained in at most one proposed spanning tree and
- each edge in *Z* is contained in at most $2n+5$ of the proposed spanning trees.

From these observations, it is easy to conclude that *T* is the unique maximum spanning tree w.r.t. the edge weights $\omega (e)$, i.e., the only candidate for a Condorcet tree (see Lemma 1). Note that

- for each $h\in A_{H}$ we have $|T\setminus T_{h}|=0$,
- for each $\gamma \in A_{C}$ it holds that $|T\setminus T_{\gamma }|=|H|=n$,
- for each $\zeta _{t,q}\in A_{Z}$ we have $|T\setminus T_{\zeta _{t,q}}|=|(B\cup H)\setminus (B\setminus \{b_{t}\})|=|H\cup \{b_{t}\}|=n+1$, and
- for each $\delta \in A_{D}$, $|T\setminus T_{\delta }|=|H|=n$ holds.

*Step II: Proving the reduction.* We complete the proof by showing that the following claim holds:

$\mathcal {U}$ is a “yes”-instance of 3-Unsat if and only if $\mathcal {V}$ is a “yes”-instance of Condorcet-Tree.

“$\underline{\Leftarrow }$”: Let $\mathcal {V}$ be a “yes”-instance of Condorcet-Tree. As a consequence, *T* is the unique Condorcet tree. Assume that $\mathcal {U}$ is a “no”-instance of 3-Unsat, i.e., there is a truth assignment $\phi$ that satisfies all clauses in *C*. We show that this leads to the conclusion that *T* is not a Condorcet tree in $\mathcal {V}$ and hence to a contradiction.

Recall that $|\phi |=n$ and consider the spanning tree

$$\begin{aligned} T':=\phi \cup R\cup F\cup B. \end{aligned}$$

We can derive the following conclusions concerning the agents in *A*:

- agents in $A_{C}$: for each $\gamma _{j}$ we have $|T'\setminus T_{\gamma _{j}}|=|\phi \setminus \{z_{j_{1}},z_{j_{2}},z_{j_{3}}\}|\le n-1$, since the satisfying truth assignment $\phi$ contains at least one literal of the clause $C_{j}$. Thus, for each $\gamma \in A_{C}$ we have $|T'\setminus T_{\gamma }|<|T\setminus T_{\gamma }|$.
- agents in $A_{Z}$: for each $1\le t\le n$, we get
  $$\begin{aligned} \begin{array}{ccl} |T'\setminus T_{\zeta _{t,q}}| &{} = &{} |(\phi \cup B)\setminus (\{x_{t},\bar{x}_{t}\}\cup B\setminus \{b_{t}\})|\\ &{} = &{} |\{b_{t}\}\cup (\phi \setminus \{x_{t},\bar{x}_{t}\})|\\ &{} = &{} 1+(n-1)\\ &{} = &{} n \end{array} \end{aligned}$$
  because per definition $\phi$ contains exactly one of $\{x_{t},\bar{x}_{t}\}$ for each *t*. Hence, for each $\zeta \in A_{Z}$ we have $|T'\setminus T_{\zeta }|<|T\setminus T_{\zeta }|$.
- agents in $A_{D}$: for each $\delta \in A_{D}$, $|T'\setminus T_{\delta }|=|\phi |=n$ holds. Thus, for each $\delta \in A_{D}$ we have $|T'\setminus T_{\delta }|=|T\setminus T_{\delta }|$.

On the other hand, the agents in $A_{H}$ propose the spanning tree *T*. Hence, we get

$$\begin{aligned} \#\{a\in A:|T\setminus T_{a}|<|T'\setminus T_{a}|\}=|A_{H}|=m+2n^{2}+n \end{aligned}$$

and

$$\begin{aligned} \#\{a\in A:|T'\setminus T_{a}|<|T\setminus T_{a}|\}=|A_{C}|+|A_{Z}|=m+2n^{2}+n. \end{aligned}$$

Thus, *T* is not a Condorcet tree. Hence there is no Condorcet tree in $\mathcal {V}$, which contradicts our assumption. As a consequence, $\mathcal {U}$ must be a “yes”-instance of 3-Unsat.

“$\underline{\Rightarrow }$”: Let $\mathcal {V}$ be a “no”-instance of Condorcet-Tree. Thus, *T* is not a Condorcet tree, i.e., there must be a spanning tree $\hat{T}\not =T$ such that

3 $$\begin{aligned} \#\{a\in A:|T\setminus T_{a}|<|\hat{T}\setminus T_{a}|\}\le \#\{a\in A:|\hat{T}\setminus T_{a}|<|T\setminus T_{a}|\} \end{aligned}$$

holds. Clearly, all $a\in A_{H}$ have $|T\setminus T_{a}|<|\hat{T}\setminus T_{a}|$. Hence, (3) implies

4 $$\begin{aligned} \#\{a\in A:|\hat{T}\setminus T_{a}|<|T\setminus T_{a}|\}\ge |A_{H}|. \end{aligned}$$

The proof proceeds in four steps.

(i) *First, we show that we can assume that*$\hat{T}\cap H=\emptyset$. Assume $\hat{T}\cap H\not =\emptyset$. Create the spanning tree $T'$ of *G* from $\hat{T}$ by replacing each edge $h_{t}\in H$ by an edge $e\in \{f_{t,0},x_{t}\}$ which (a) is not included in $\hat{T}$ and (b) satisfies that $\hat{T}\cup \{e\}\setminus \{h_{t}\}$ is in fact a spanning tree of *G*. Obviously, $|T\setminus T_{a}|<|T'\setminus T_{a}|$ and $|T\setminus T_{a}|<|\hat{T}\setminus T_{a}|$ hold for $a\in A_{H}$. However, since there is no tree proposed by an agent in $A\setminus A_{H}$ which contains an edge in *H*, we have

$$\begin{aligned} |T'\setminus T_{a}| \le |\hat{T}\setminus T_{a}| \end{aligned}$$

for $$a\in A\setminus A_{H}$$. Hence,

$$\begin{aligned} \#\{a\in A:|T\setminus T_{a}|<|T'\setminus T_{a}|\}\le \#\{a\in A:|T'\setminus T_{a}|<|T\setminus T_{a}|\} \end{aligned}$$

holds as well.

(ii) *Second, we show that*$(R\cup B\cup F)\subset \hat{T}$*holds.* By construction, $R\subset \hat{T}$. Let $\kappa :=|(B\cup F)\setminus \hat{T}|$. Assume that $\kappa \ge 1$ holds.

If $\kappa >n$, then each $\delta \in A_{D}$ has $|T\setminus T_{\delta }|=n<\kappa \le |\hat{T}\setminus T_{\delta }|$, because $[(B\cup F)\setminus \hat{T}]\subseteq (T_{\delta }\setminus \hat{T})$ and $|T_{\delta }\setminus \hat{T}|=|\hat{T}\setminus T_{\delta }|$ hold. Thus,

$$\begin{aligned} \begin{array}{ccl} \#\{a\in A:|T\setminus T_{a}| < |\hat{T}\setminus T_{a}|\} &{} \ge &{} |A_{H}|+|A_{D}|\\ &{} = &{} (m+2n^{2}+n)+4n+1\\ &{} > &{} \frac{|A|}{2} \end{array} \end{aligned}$$

because the total number of agents is $|A|=2m+4n^{2}+6n+1$ (stated in (2)). This contradicts (3).

Hence, $\kappa \le n$ must hold. However, $\hat{T}$ can have a non-empty intersection with at most 2*n* from the sets $D_{m+2n^{2}+n+i}$, $1\le i\le 4n+1$, because otherwise $\hat{T}$ contains at least

$$\begin{aligned} \begin{array}{ccl} |R|+(|B\cup F|-\kappa )+(2n+1) &{} = &{} n+(n+n(M+1)-\kappa )+(2n+1)\\ &{} = &{} nM+5n+1-\kappa \\ &{} \ge &{} nM+4n+1 \end{array} \end{aligned}$$

edges, which contradicts (1). Hence, for at least $2n+1$ agents $\delta _{i}\in A_{D}$ — i.e., for at least $2n+1$ of the indices $i\in \{1,\ldots ,4n+1\}$) — we have $|\hat{T}\setminus T_{\delta _{i}}|=|T_{\delta _{i}}\setminus \hat{T}|=\kappa +|D_{m+2n^{2}+n+i}|\ge 1+n$. Since $|T\setminus T_{\delta }|=n$ holds for each $\delta \in A_{D}$,

$$\begin{aligned} \begin{array}{ccl} \#\{a\in A:|T\setminus T_{a}| < |\hat{T}\setminus T_{a}|\} &{} \ge &{} |A_{H}|+2n+1\\ &{} = &{} (m+2n^{2}+n)+2n+1\\ &{} > &{} \frac{|A|}{2} \end{array} \end{aligned}$$

follows. Again this contradicts (3). Thus, $\kappa =0$, i.e., $(B\cup F)\subset \hat{T}$ and hence $(R\cup B\cup F)\subset \hat{T}$ holds.

(iii) *Third, we show that*$|\{x_{t},\bar{x}_{t}\}\cap \hat{T}|=1$*holds for each*$1\le t\le n$. From $B\subset \hat{T}$ (see (ii)), it follows that $|\{x_{t},\bar{x}_{t}\}\cap \hat{T}|\le 1$ holds for each $1\le t \le n$, because otherwise $\hat{T}$ would contain a cycle which contradicts with the fact that $\hat{T}$ is a spanning tree. Assume that for some *t*, $|\{x_{t},\bar{x}_{t}\}\cap \hat{T}|=0$ holds. By (ii), Eq. (1) implies that $\hat{T}$ can have a non-empty intersection with at most *n* from the sets $D_{m+(t-1)(2n+1)+q}$, $1\le q\le 2n+1$. Thus, for at least $n+1$ indices $q\in \{1,\ldots ,2n+1\}$ we have

$$\begin{aligned} |T_{\zeta _{t,q}}\setminus \hat{T}|= & {} |\{x_{t},\bar{x}_{t}\}\cup \{D_{m+(t-1)\cdot (2n+1)+q}\setminus \{d_{t,m+(t-1)\cdot (2n+1)+q}\}|\\= & {} 2+(n-1)\\= & {} n+1. \end{aligned}$$

Recalling that $|\hat{T}\setminus T_{\zeta _{t,q}}|=|T_{\zeta _{t,q}}\setminus \hat{T}|$ holds, we hence can conclude that $|\hat{T}\setminus T_{\zeta _{t,q}}|=|T\setminus T_{\zeta _{t,q}}|$ is satisfied for at least $n+1$ agents in $A_{Z}$.

Analogously, $\hat{T}$ can have a non-empty intersection with at most *n* from the sets $D_{m+2n^{2}+n+i}$, $1\le i\le 4n+1$. Thus, at most *n* of the voters $\delta \in A_{D}$ have $|\hat{T}\setminus T_{\delta }|< n$ and hence $|\hat{T}\setminus T_{\delta }| < |T\setminus T_{\delta }|$. As a consequence, we get

$$\begin{aligned} \#\{a\in A:|T\setminus T_{a}|<|\hat{T}\setminus T_{a}|\}\ge |A_{H}|=m+2n^{2}+n \end{aligned}$$

while

$$\begin{aligned} \#\{a\in A:|\hat{T}\setminus T_{a}|<|T\setminus T_{a}|\}\le & {} |A_{C}|+(|A_{Z}|-(n+1))+n\\= & {} m+(2n^{2}+n-n-1)+n\\= & {} m+2n^{2}+n-1. \end{aligned}$$

Therewith,

$$\begin{aligned} \#\{a\in A:|T\setminus T_{a}|<|\hat{T}\setminus T_{a}|\}>\#\{a\in A:|\hat{T}\setminus T_{a}|<|T\setminus T_{a}|\} \end{aligned}$$

holds, which contradicts (3).

(iv) *Finally, from*$\hat{T}$*we derive a truth assignment*$\phi$*that satisfies all clauses inC*. Let $\phi :=Z\cap \hat{T}$. From (iii), we know that for each $1\le t\le n$, exactly one of $\{x_{t},\bar{x}_{t}\}$ is contained in $\phi$. In other words, $|\phi |=n$ holds and $\phi$ is a feasible truth assignment. In addition, (iii) implies that $(D\cup H)\cap \hat{T}=\emptyset$ holds because otherwise (1) would be violated. Hence, we can write $\hat{T}$ as $\hat{T}=R\cup B\cup F\cup \phi$. Consider the agents in $A\setminus A_{C}$:

- for each agent $h\in A_{H}$ we have $|\hat{T}\setminus T_{h}|=n$, whereas $|T\setminus T_{h}|=0$; i.e., $|T\setminus T_{h}|<|\hat{T}\setminus T_{h}|$.
- for each agent $\zeta _{t,q} \in A_{Z}$ we have $|\hat{T}\setminus T_{\zeta _{t,q}}|= |(\phi \setminus \{x_t,\bar{x}_t\})\cup \{b_t\}|=(n-1)+1=n$, whereas $|T\setminus T_{\zeta _{t,q}}|=1+n$; i.e., $|\hat{T}\setminus T_{\zeta _{t,q}}|<|T\setminus T_{\zeta _{t,q}}|$.
- for each agent $\delta \in A_{D}$ we have $|T\setminus T_{\delta }|=|\hat{T}\setminus T_{\delta }|=n$.

As a result, due to $|A_{H}|=m+2n^{2}+n$ and $|A_{Z}|=2n^{2}+n$, in order to satisfy (3) each agent $\gamma \in A_{C}$ must have $|\hat{T}\setminus T_{\gamma }|<|T\setminus T_{\gamma }|=n$. Because of $D\cap \hat{T}=\emptyset$, the only possibility to achieve this is that, for each $1\le j\le m$, at least one of the edges $\{z_{j_{1}},z_{j_{2}},z_{j_{3}}\}$ is contained in $\hat{T}$. In other words, for each $1\le j\le m$, at least one of the variables $\{z_{j_{1}},z_{j_{2}},z_{j_{3}}\}$ that make up clause $C_{j}$ must be contained in, i.e., set true under $\phi$. Thus, $\phi$ satisfies all clauses in *C*, i.e., $\mathcal {U}$ is a “no”-instance of 3-Unsat. $\square$

In the following theorem, we state an analogous result for Weak-Condorcet-Tree.

### **Theorem 2**

Weak-Condorcet-Tree is $\mathsf {coNP}$-hard.

### *Proof*

The proof follows from the one of Theorem 1 by removing exactly one agent from the set $A_{H}$.$\square$

We now turn to the problems of deciding whether a given spanning tree is a (weak) Condorcet tree. These problems turn out to be $\mathsf {coNP}$-complete.

### **Theorem 3**

Condorcet-Tree Winner is $\mathsf {coNP}$-complete.

### *Proof*

In the proof of Theorem 1, there is exactly one candidate for a Condorcet tree in instance $\mathcal {V}$, i.e., the spanning tree *T*. Thus, in instance $\mathcal {V}$ the answer to question (Q1) “Is there a Condorcet tree?” is “Yes” if and only if the answer to (Q2) “Is *T* a Condorcet tree?” is “Yes”. It is hence straightforward that Condorcet-Tree Winner is $\mathsf coNP$-hard.

Finally, membership in $\mathsf coNP$ is easy to verify. Let $\mathcal {C}$ be an instance of Condorcet-Tree Winner, where we ask if $T_\mathcal {C}$ is a Condorcet tree. If $\mathcal {C}$ is a “no”-instance, then any tree $T'$ which satisfies $\#\{a\in A:|T_\mathcal {C}\setminus T_{a}|<|T'\setminus T_{a}|\} \le \#\{a\in A:|T'\setminus T_{a}|<|T_\mathcal {C}\setminus T_{a}|\}$ serves as certificate. $\square$

Analogously, with (the proof of) Theorem 2 we get the following result.

### **Theorem 4**

Weak-Condorcet-Tree Winner is $\mathsf {coNP}$-complete.

## Conclusion

We have shown that both deciding if a (weak) Condorcet tree exists and deciding if a given spanning tree is a (weak) Condorcet tree, are computationally difficult problems. While the latter problems turn out to be $\mathsf {coNP}$-complete, our $\mathsf {coNP}$-hardness results for Condorcet-Tree and Weak-Condorcet-Tree only provide lower bounds for their computational complexity. A finer placement of these problems in terms of their computational complexity is an interesting open question.

## Footnotes

1 Respectively, in its weaker version, does not lose by a simple majority against any other alternative in a pairwise comparison

2 In this context, “popular tree” translates into “weak Condorcet tree” in our setting.

3 From a technical viewpoint, note that the main proofs in Darmann (2013b, 2014) reduce from variants of 2-Sat, while in our main proof construction we provide a reduction from 3-Unsat.
